# The evolving role of open abdominal surgery in gynecological malignancies in the era of minimally invasive and precision oncology

**DOI:** 10.1186/s12957-026-04510-4

**Published:** 2026-09-07

**Authors:** Miklos Acs, Veronika Müller, Ulrich Kaiser, Clemens Tempfer, Björn Lampe

**Affiliations:** 1 Department of Surgery, University Hospital Regensburg, Regensburg, Germany; 2 ÜBAG Dr. Vehling-Kaiser MVZ GmbH, Landshut, Germany; 3 Department of Gynecology, University Hospital Ruhr-Bochum, Bochum, Germany; 4 Department of Gynecology, Städtische Kliniken Mönchengladbach, Mönchengladbach, Germany

**Keywords:** Gynecological malignancies, Open abdominal surgery, Laparotomy, Minimally invasive surgery, Robotic surgery, Cytoreductive surgery, Cervical cancer, Ovarian cancer, Endometrial cancer, Surgical oncology

## Abstract

**Background:**

Oncological surgery is undergoing profound transformation driven by advances in systemic therapy, immunotherapy, and minimally invasive techniques. This narrative review examines whether open abdominal surgery retains clinical relevance in gynecological malignancies or has been superseded by modern alternatives.

**Methods:**

A literature search was conducted in PubMed combining terms related to gynecological malignancies, abdominal surgical procedures (open, laparoscopic, robotic, cytoreduction, pelvic exenteration), and personalized systemic therapies. Original articles, reviews, and guidelines in German and English were included. Two independent reviewers screened titles, abstracts, and full texts, extracting data on study design, interventions, and clinical endpoints (R0 resection, morbidity, progression-free and overall survival).

**Results:**

For cervical cancer, open radical abdominal hysterectomy as the standard of care was internationally confirmed. In endometrial cancer, minimally invasive approaches are established for early-stage disease, while laparotomy remains the gold standard in advanced stages requiring cytoreductive surgery. For ovarian, tubal, and primary peritoneal carcinoma, open cytoreductive surgery with the goal of macroscopically complete resection remains guideline recommended. Emerging biomarkers such as circulating tumor DNA may further refine patient selection and surgical decision-making.

**Conclusion:**

Open abdominal surgery remains indispensable in gynecological oncology. Rather than being replaced, it is evolving into a specialized, biology-adapted component of multimodal treatment strategies. Surgical indications are becoming less frequent but more complex and individualized. Patient selection in experienced centers remains the decisive factor.

## Background

Oncological abdominal surgery is radically changing. Advances in systemic therapy, immunotherapy, and molecular medicine are leading to a reassessment of traditional surgical indications [1–3]. At the same time, minimally invasive and robotic techniques have revolutionized surgical procedures [4]. While surgery is becoming less prominent or is being replaced by de-escalation strategies in the early stages of certain tumors, it is gaining importance in complex cases. Surgical tumor specimens also provide the basis for further molecular analyses and biomarker testing, and thus for increasingly targeted systemic therapy in the context of “precision oncology.” This article examines the role of abdominal surgery in the context of personalized medicine and minimally invasive surgical techniques. The central question is: Will traditional abdominal surgery be replaced by these innovations, or will it evolve into an integral, specialized component of a multimodal treatment approach? This question will be discussed with a particular focus on gynecological malignancies.

## Methods

For this narrative review, in January 20,206 a broad literature search was conducted in the scientific database PubMed. The search combined terms and free text related to gynecological malignancies, abdominal surgical procedures (open, laparoscopic, robotic, cytoreduction, pelvic exenteration), and personalized systemic therapy (precision medicine, biomarkers including circulating tumor deoxyribonucleic acid (DNA), minimal residual disease [ctDNA/MRD], Poly [ADP-ribose] polymerases [PARP]/checkpoint inhibition). Example search strings employed were: for cervical cancer (“cervical cancer” OR “cervical carcinoma”) AND (“laparotomy” OR “open surgery” OR “radical hysterectomy” OR “minimally invasive”); for ovarian cancer (“ovarian cancer” OR “tubal carcinoma” OR “peritoneal carcinoma”) AND (“cytoreductive surgery” OR “debulking” OR “laparoscopy” OR “HIPEC”); and for endometrial cancer (“endometrial cancer” OR “uterine cancer”) AND (“laparoscopy” OR “laparotomy” OR “robotic surgery” OR “molecular classification”). Both national and international human studies in German and English (original articles, reviews, guidelines) published between January 2009 and January 2026 with a surgical focus and clinical endpoints (R0, morbidity, progression free survival/overall survival [PFS/OS]) were included. Grey literature and conference abstracts were not considered. Two experienced reviewers (MA, BL) screened titles and abstracts, evaluated full texts, and extracted study design, population, interventions, outcomes, and biomarker data. Due to the narrative design of this review no formal risk-bias-tool was applied. However, the level of evidence of the key trials is highlighted.

## Results

### Cervical cancer

According to current guidelines, a radical hysterectomy and locoregional lymphadenectomy are recommended for most patients with cervical cancer at Fédération Internationale de Gynécologie et d’Obstétrique (FIGO) stages IA2, IB1–3, and IIA. Primary chemoradiotherapy, on the other hand, is recommended for locally advanced disease (FIGO IIB-IVA) and, in more recent approaches, is also combined with induction chemotherapy or immunotherapy with pembrolizumab [1, 3, 5]. However, surgery remains part of the treatment plan even in cases of locally advanced cervical cancer, as surgical staging, involving a systematic pelvic and, if necessary, para-aortic lymphadenectomy, is performed prior to chemoradiotherapy in order to precisely determine the stage of the disease and define the radiation field [6]. In the context of surgical treatment for cervical cancer, minimally invasive techniques such as laparoscopy and robotic surgery have been increasingly used alongside vaginal and abdominal surgery since the early 2000s. The advantage of these techniques lies in lower overall peri- and postoperative morbidity and a shorter recovery period. However, neither laparoscopic nor robotic surgery offers a survival benefit [6]. On the contrary, one of the most frequently cited studies of the past 10 years on cervical cancer, the Laparoscopic Approach to Cervical Cancer (LACC) study, demonstrated that patients with cervical cancer who underwent minimally invasive radical hysterectomy had significantly poorer recurrence-free survival and overall survival [7]. Specifically, this large, randomized study, consistent with epidemiological data [8], demonstrated that in FIGO stages IA1 (with lymphatic invasion), IA2, and IB1 (tumor diameter ≤ 4 cm), open laparotomy is superior to minimally invasive (conventional laparoscopic or robotic) surgery. Following minimally invasive surgery (MIS), the LACC trial demonstrated significantly inferior disease-free survival compared to open surgery in these tumor stages (85% MIS vs. 96% open; Hazard Ratio [HR] 3.9; 95% Confidence Interval [CI] 2–7.6), meaning that women undergoing MIS had a 3.9-fold higher hazard of recurrence or death. Likewise, 4.5-year overall survival was significantly lower after MIS compared with open surgery (91% vs. 96%; HR 2.7; 95% CI 1.3–5.6) [7]. The difference in survival was evident both in the overall cohort and among patients with tumors < 2 cm and ≥ 2 cm (HR 4.24; 95% CI 1.7–10.4) and was also observed in the 4.5-year analysis of the LACC study in all subgroups examined, with the exception of patients who had undergone conization [7]. However, some limitations of this study must be considered. The experience and case volume of surgeons was not measured. Therefore, a learning-curve effect could bias the results. Additionally at the time of this trial colpotomizers and closed-cuff techniques were not yet introduced, potentially influencing the tumor spillage. Both MIS and preoperative imagining were not standardized within the trial, leading to the potential of selection bias and casual interfering Still, a meta-analysis showed similar results, with a higher risk of recurrence or death associated with minimally invasive radical hysterectomy, for both laparoscopic and robotic procedures (HR 1.71; 95% CI 1.36–2.15) [9]. This surprising finding from the LACC study led to an international revision of clinical guidelines, such as the German S3 guideline and the National Comprehensive Cancer Network (NCCN) guideline. Marking a return to open radical abdominal hysterectomy as the recommended standard procedure [6, 10, 11]. However, several clinical trials are currently evaluating safety measures to determine whether laparoscopic radical hysterectomy is possible without compromising oncological outcomes [12–15]. In patients with advanced cervical cancer (FIGO stages IIB–IVA) and in patients with node- disease, primary radical hysterectomy is not indicated. As adjuvant chemoradiotherapy is necessary in these cases, radical hysterectomy offers no additional therapeutic benefit, and the combination of chemoradiotherapy and radical hysterectomy is associated with an increased morbidity rate [2, 6]. Whether this approach is associated not only with lower morbidity but also with equivalent survival rates has not yet been demonstrated in randomized trials. However, a large, international retrospective data analysis, the ABandoning RAdical hysterectomy in cerviX cancer (ABRAX) study, shows that radical hysterectomy does not offer any survival benefit when added to primary chemoradiotherapy [16]. Currently, radical hysterectomy is not performed in advanced stages of the disease. Instead, primary chemoradiotherapy is administered following minimally invasive surgical staging (systematic pelvic and, if necessary, para-aortic lymphadenectomy) [2, 6, 17]. In addition, the use of the Programmed cell death protein (PD)-1 checkpoint inhibitor pembrolizumab is recommended for patients with certain characteristics [17] (Spread of the tumor to the pelvic wall, involvement of the lower third of the vagina, hydronephrosis or renal failure resulting from tumor spread and involvement of the bladder or rectum, as well as tumor infiltration beyond the pelvis).

Following treatment for early-stage cervical cancer, distant metastases or recurrences develop in 15% to 60% of cases. This typically occurs within the first two years after completion of treatment [2, 6]. In most cases, metastatic cervical cancer is incurable. In selected patients with pelvic recurrence (locoregional recurrence) or limited distant metastasis, radical exenteration may be a potentially curative surgical treatment [6, 18]. Eralp et al. defined clear criteria for patient selection in this context [19]:

- A central pelvic recurrence without fixation to the sidewall or associated hydronephrosis
- A long disease-free interval
- A tumor diameter of less than 3 cm in the recurrence.

In contrast, other authors argue that infiltration of the pelvic wall is no longer an absolute contraindication. Depending on the extent of tumor spread, a laterally extended en bloc resection including vessels, nerve structures, and musculature, up to and including partial sacrectomy, may be considered, if this allows for complete tumor resection in the sense of an R0 resection [20].

### Endometrial cancer

The standard surgical procedure for endometrial cancer is total extrafascial hysterectomy with bilateral salpingo-oophorectomy [21, 22]. In the early stages, procedures should be performed using minimally invasive techniques. Other procedures (e.g., laparotomy, vaginal) are selected on an individual basis based on patient- and tumor-specific factors. Randomized studies have shown that minimally invasive procedures result in lower peri- and postoperative complication rates, less pain, and a shorter recovery time than laparotomies, without negatively affecting long-term oncological outcomes [23, 24]. The Laparoscopic Approach to Carcinoma of the Endometrium (LACE) trial confirmed long-term non-inferiority of laparoscopic versus open surgery for early-stage endometrial cancer, with no significant difference in recurrence-free or overall survival at five-year follow-up [25]. Similarly, the Laparoscopy Study 2 by the Gynecologic Oncology Group (GOG LAP2 trial) demonstrated that laparoscopic staging for uterine cancer resulted in fewer complications and shorter hospital stay without compromising oncological outcomes, providing level I evidence supporting the current MIS-first recommendation for early-stage disease [24].

Robotic procedures have proven to be particularly advantageous. For example, a meta-analysis of 33 studies comparing surgical outcomes in patients with endometrial cancer found that robotic surgery, compared to conventional laparoscopy, was associated with a shorter hospital stay (mean difference − 0.46; 95% CI -0.62 to -0.24), lower blood loss, and lower conversion rates (risk ratio 0.41; 95% CI 0.29–0.59) [26]. While there were fewer adverse events overall in the robotic group, the financial cost was significantly higher. These findings are also reflected in current national and international guidelines. Accordingly, minimally invasive surgery (conventional laparoscopic or robotic) is unanimously recommended as the standard of care [10, 21]. However, some authors and institutions, such as the U.S. Food and Drug Administration (FDA), emphasize that there is currently a lack of safety data on robotic surgery for endometrial cancer [27]. Although there is currently no general consensus regarding the best approach for assessing locoregional tumor spread in lymph nodes (sentinel lymph node biopsy vs. conventional lymph node dissection) due to the lack of prospective randomized trials, the current German S3 guideline, as well as those of the NCCN and the Society of Gynecologic Oncology (SGO) support the role of sentinel lymph node biopsy (SLN) in patients with endometrial cancer [10, 21, 28].

For patients with advanced-stage cancer, laparotomy remains the gold standard [29]. According to current S3 guidelines, cytoreductive surgery may be performed in cases of locally advanced disease with or without locoregional metastasis if it is highly likely that a resection leaving no residual tumor is possible [21]. This underscores the continued value of complex surgery for patients with advanced but potentially curable endometrial cancer.

Regarding precision oncology the The Cancer Genome Atlas project (TCGA ) identified four molecular subgroups of endometrial carcinoma (POLE-ultra-mutated, mismatch repair-deficient (dMMR/MSI-H), p53-mutated (copy number high), and no specific molecular profile (NSMP)) [30], With each of these subtypes influencing the prognosis and response to adjuvant therapies [31] and immunotherapy [32, 33]. Hereby surgery has a subordinate role as the molecular classification can be performed on pre-operative tissue samples [34].

### Tubal carcinoma, ovarian carcinoma, and primary peritoneal carcinoma

For tubal carcinoma, ovarian carcinoma, and primary peritoneal carcinoma, which should be considered a single biological and therapeutic entity, open abdominal surgery, specifically laparotomy with cytoreductive surgery, represents the standard approach for surgical treatment. Minimally invasive surgery using conventional and robot-assisted laparoscopy has been studied primarily in patients with suspected stage I or II ovarian cancer who do not require cytoreduction [35, 36]. An analysis of the National Cancer Database showed that, among patients with FIGO Stage I ovarian cancer, the conversion rate was lower with robotic surgery than with conventional laparoscopy (conversion rate of 7.2% versus 17.9%). The survival rate was comparable for both procedures [37]. Compared to laparotomy, minimally invasive surgery offers the advantages of less blood loss, a shorter hospital stay, fewer postoperative complications, and a faster recovery. However, minimally invasive surgery is associated with a higher risk of ovarian tumor rupture, which could potentially worsen the prognosis [38, 39]. In addition, there is an increased rate of port-site metastases [40].

In advanced stages of the disease, surgical cytoreduction aimed at achieving macroscopically complete tumor-free status is the treatment of choice. However, the use of minimally invasive surgery for cytoreduction is increasing. In an analysis of the National Cancer Database of 7,897 patients with advanced ovarian cancer who underwent interval cytoreduction following neoadjuvant chemotherapy, 25% of patients underwent minimally invasive surgery. These patients had an improved overall survival rate compared to those who underwent laparotomy (47 versus 41 months; HR 0.86; 95% CI 0.79–0.94) [41]. Several recent studies show no difference between laparotomy and minimally invasive surgery in terms of achieving an optimal resection [9, 41]. (Table 1). Current national and international guidelines, however, still do not recognize minimally invasive surgery as a safe procedure for cytoreduction [42, 43]. Specifically, the current S3 guideline recommends laparotomy as the standard approach for primary, interval, and recurrent surgery, and assigns laparoscopy only a role as a diagnostic tool [43]. The NCCN does not provide a clear recommendation for or against the use of minimally invasive surgery [44].

**Table 1:** Study overview comparing open surgery and minimally invasive surgery (MIS)

Nr.	Author (year)	Journal	Study Type/ Level of Evidence	Topic / Method	*n*	Results	Key Limitations	Rate of Conversion to Open Surgery
1	Rauh-Hain JA et al., 2024 [45]	JAMA Netw Open	RCT/ Level I	LANCE Trial – MIS vs. Laparotomy IDS	100	R0 88% vs. 83%, No superiority, guaranteed feasibility	Likely underpowered due to small sample size	12.5% (6/48 surgeries)
2	Jørgensen K et al., 2023 [41]	Gynecol Oncol	Cohort/ Level II	MIS IDS vs. open	143	Similar PFS/OS, lower morbidity with MIS	No randomization	10.3% (208/2021 surgeries)
3	Ceccaroni M et al., 2023 [46]	Cancers (Basel)	Retrospective/ Level III	Laparoscopic PDS/IDS/SDS	108	R0 > 95%, 19% complications	No comparison to open surgery, relatively high complication rate (19%)	Not stated
4	Conte C et al., 2023 [47]	Int J Gynecol Cancer	Retrospective/ Level III	Minimal invasive vs. secondary Cytoreduction	82	Possible in selective cases	Limited to selective cases	Not stated
5	Uwins C et al., 2024 [48]	Int J Gynecol Cancer	Prospective / Level II	MIRRORS – robotic IDS	56	R0 > 90%, low morbidity	Small sample size, no open surgery control	0% (0/20 surgeries)
6	Van Trappen P et al., 2023 [49]	Eur J Obstet Gynecol Reprod Biol	Comparison / Level III	Robotic surgery vs. open in early and advanced stages of ovarian cancer	96	Similar outcome in early stages	Mixture of early and advanced stage patients	Not stated
7	Hayek J et al., 2025 [50]	J Clin Med	Retrospective / Level III	Robotic IDS (2010–2019)	112	No difference in survival to open surgery	Long inclusion period, learning curve effect	Not stated

*Abbreviations*: *RCT* Randomized controlled trial, *IDS* Interval Debulking Surgery, *PDS* Primary Debulking Surgery, *SDS* Secondary Debulking Surgery, *ROC* Recurrent Ovarian Cancer, *LOS* Length of Stay

Several additional landmark trials warrant discussion in the context of advanced ovarian cancer surgery. The Lymphadenectomy in Ovarian Neoplasms LION trial [51] demonstrated that systematic pelvic and para-aortic lymphadenectomy does not improve survival in patients with advanced ovarian cancer who achieve macroscopically complete cytoreduction (R0), despite markedly higher complication rates in the lymphadenectomy group. This finding has refined the surgical approach by permitting de-escalation of nodal dissection in selected patients with R0 resection, without compromising oncological outcomes [51]. The German Endometrial and Ovarian Cancer Therapy Optimization Program - DESKTOP III trial established that secondary cytoreductive surgery in platinum-sensitive recurrent ovarian cancer confers a significant overall survival benefit (HR 0.76, 95% CI 0.59–0.97) when patients are selected using the validated score of the Working Group on Gynecologic Oncology (Arbeitsgemeinschaft Gynäkologische Onkologie, AGO): positive ECOG performance status, ascites-free complete resection at first surgery, and absence of residual tumor. Providing level I evidence for open surgery in the recurrence setting [52]. The Surgical Complications Overalled Randomized Protocol in Interval versus Optimal Neo-adjuvant treatment (SCORPION) trial raised important questions about the benefit of aggressive primary cytoreductive surgery versus neoadjuvant chemotherapy followed by interval debulking in molecularly high-risk patients, supporting individualized surgical decision-making rather than a uniform aggressive upfront approach [53].

Promising developments are emerging in the detection of ctDNA in the blood for determining prognosis, MRD, and monitoring treatment response. This method offers a potential new biomarker for personalized, interdisciplinary surgical decision-making [54, 55]. This could, for example, make it easier to decide whether further surgery, a second-look procedure, additional therapy, or close monitoring is appropriate. For example, a high ctDNA after chemotherapy could serve as an indicator for maximally aggressive open cytoreductive surgery, as a high circulating tumor burden signals incomplete systemic disease control. Conversely, ctDNA-guided surveillance offers the possibility of detecting early recurrence amenable to secondary debulking surgery before clinically manifest progression. In the future, MRD negativity confirmed by ctDNA assay may become a criterion to consider de-escalation from open to minimally invasive completion surgery in selected patients who have achieved deep molecular remission. Especially in patients at high risk of recurrence, ctDNA monitoring could help detect minimal residual disease at an early stage, thereby enabling personalized management [54].

As ovarian cancer is prone to peritoneal metastases [56, 57] adequate tools to combat this difficult tumor manifestation are necessary. Hereby Cytoreductive Surgery (CRS) followed by Hyperthermic Intraperitoneal Chemotherapy (HIPEC) plays a central role. With studies such as the Ovarian Hyperthermic Intraperitoneal Chemotherapy (OVHIPEC-1) trial [56] have demonstrated a statistically significant improvement in both recurrence-free survival (14.2 vs. 10.7 months; HR 0.66, 95% CI 0.50–0.87) and overall survival (45.7 vs. 33.9 months; HR 0.67, 95% CI 0.48–0.94). The CHIPOR trial (Chimio-Hyperthermie Intra-Péritonéale d’Ovaires en Récidive (Intraperitoneal Hyperthermic Chemotherapy for Recurrent Ovaries)) even demonstrated a survival benefit attributed to HIPEC in patients with recurrent disease [57]. However, the patients in this study received neoadjuvant chemotherapy prior to surgery [57]. If this was omitted, like in the HORSE/MITO-18 trial (Hyperthermic Intraperitoneal Chemotherapy in Platinum-Sensitive Recurrent Ovarian Cancer organized by the Multicenter Italian Trials in Ovarian Cancer) [58] HIPEC did not exhibited a survival benefit in patients with recurrent diseases compared to CRS alone. Crucially, HIPEC redefines the role of laparotomy: open abdominal surgery is not merely a resective tool but the indispensable delivery platform for regional intraperitoneal chemotherapy. 

**Fig. 1:**
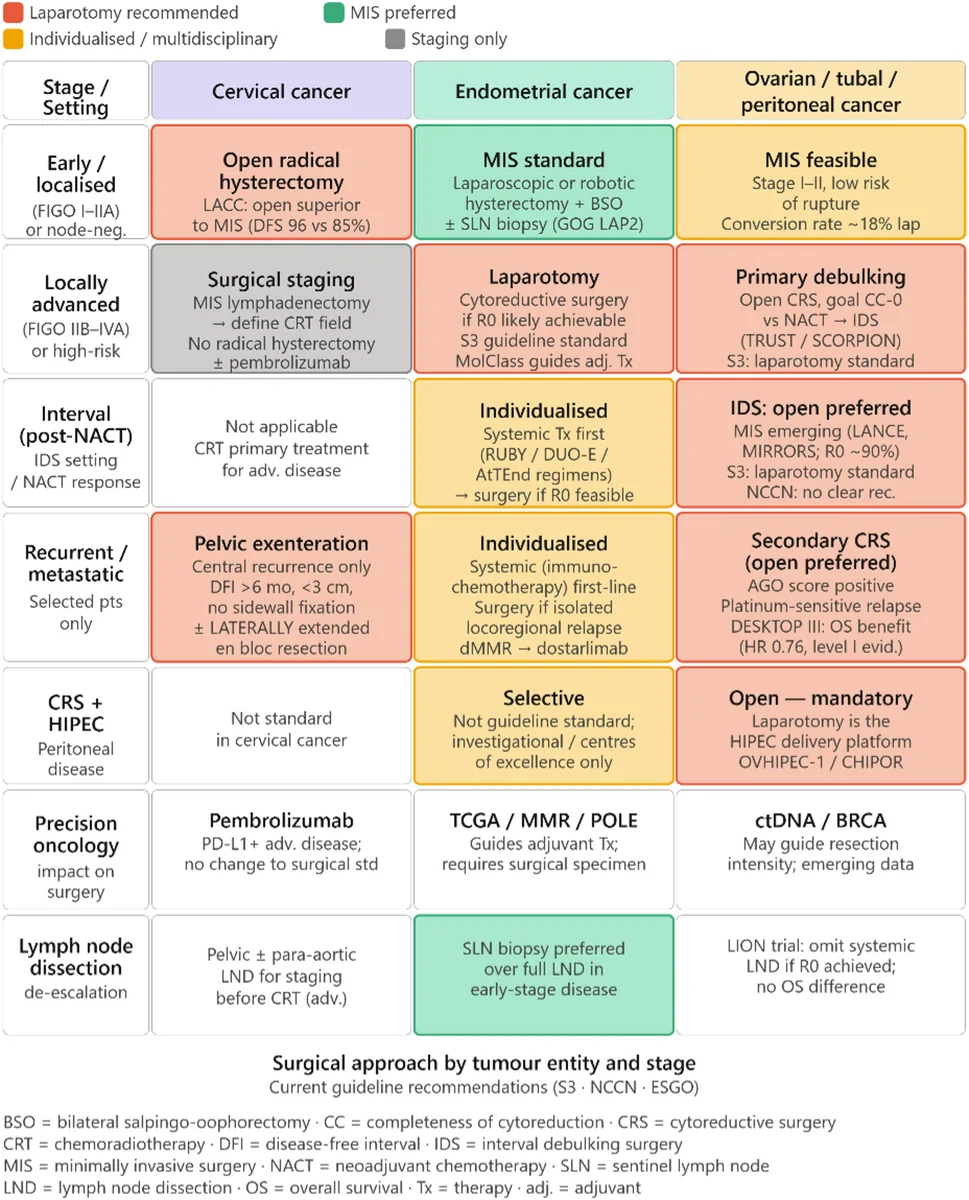
Schematic representation of guideline recommendations for the usage of MIS or open surgery for the discussed tumor entities

Finally, an overview detailing our findings regarding the guideline-recommendation for the usage of MIS or open surgery for all the examined tumor entities is given in Fig. 1.

## Discussion

The findings presented here, along with the current literature, underscore that abdominal surgery is by no means losing its importance in the age of personalized systemic therapies and minimally invasive procedures. Nevertheless, it is undergoing a significant structural transformation. While systemic therapies, targeted agents, and modern chemotherapy strategies enable far-reaching therapeutic advances in many gynecological malignancies, it is also evident that surgical interventions remain essential in both early-stage and advanced-stage situations. The evolution of the oncological treatment spectrum described in the literature confirms this shift. Surgery is becoming less frequent, but significantly more demanding and increasingly specialized or personalized.

A prime example of this is ovarian cancer, in which complete cytoreductive surgery without macroscopic tumor residue remains one of the strongest independent prognostic factors. Even though the use of neoadjuvant therapies has been intensively investigated, as in the studies Trial of Radical Upfront Surgical Therapy (TRUST) [59], Primary chemotherapy versus primary surgery for newly diagnosed advanced ovarian cancer (CHORUS) [60], or European Organisation for Research and Treatment of Cancer EORCT) 55971 [40], surgical quality remains the decisive factor. The presented data regarding laparoscopic and robotic cytoreductive surgery for ovarian cancer shows that minimally invasive procedures are technically feasible in selected situations and competitive in terms of R0 resection rates. Nevertheless, the existing literature indicates that relevant limitations exist. These include the risk of tumor rupture, port-site metastases, or technical limitations that compromise oncological outcomes. National and international guidelines continue to confirm open cytoreduction as the standard for surgical treatment of patients with ovarian cancer, tubal cancer, and primary peritoneal cancer. For these tumor entities, macroscopic complete resection (CC-0) remains the most important prognostic factor, underscoring the central role of abdominal surgery, even as neoadjuvant chemotherapy gains in importance [43].

Open surgery also remains the primary treatment for cervical cancer. Due to its oncological superiority, the LACC study [7] led to the international re-establishment of open radical hysterectomy as the standard of care; even for early-stage disease. This confirms the principle: “Not everything that is possible with minimally invasive techniques is also the right choice from an oncological standpoint.” At the same time, de-escalation approaches are emerging, such as those in the Simple Hysterectomy and Pelvic Node Assessment (SHAPE) study. It demonstrated that: in early stages of cervical cancer, once nodal negativity has been confirmed, a simple hysterectomy is not inferior to a radical hysterectomy, is oncologically safe, and significantly improves the quality of life for affected women [61]. In addition, new multimodal approaches are being explored, such as in the Induction chemotherapy followed by standard chemoradiotherapy versus standard chemoradiotherapy alone in patients with locally advanced cervical cancer (INTERLACE) study [62], which successfully tested the use of low-dose weekly induction chemotherapy to enhance primary chemoradiotherapy in advanced stages. Even with these approaches, surgery is not eliminated but is redefined as a selective, risk-adapted component. This reflects the fundamental principle: surgical indications are becoming less frequent, but more complex and individualized.

In endometrial cancer, minimally invasive procedures are becoming the new standard [10, 21]. In addition, numerous studies are investigating ways to optimize systemic therapy through the additional use of immunotherapies, particularly PD-1 and PD-L1 inhibitors. Examples of such studies include RUBY [32], DUO-E [63] and AtTEnd [64].

Despite our findings there seems to be systemic pressure to utilize MIS instead of traditional open surgery. As MIS is seen as modern and the faster recovery rates and shorter hospital are advertised, hospitals often report the utilization of these techniques and market them as the “better” option. This may inadvertently create pressure on surgeons to choose minimally invasive approaches even when open surgery is oncologically preferable. The conflict between evidence-based individualized patient decision-making and performance targets represents a genuine challenge for gynecologic oncologists and multidisciplinary tumor boards. It underscores that MIS utilization rates, in isolation, are poor proxies for surgical quality in oncology, and that patient-centered decision-making, guided by oncological evidence rather than institutional incentives, must remain paramount.

It is also important to mention that open abdominal surgery has a superior rescue capacity in the event of major complications. For example, vascular injuries to major vessels can immediately be mended in open surgery with direct compression and vascular reconstruction. Meanwhile in MIS achieving hemostasis can be difficult, often requiring time-consuming conversion to open surgery and therefore resulting in worse outcomes.

Another area of future growth in abdominal surgery lies in the integration of new technologies. Robotic systems, artificial intelligence (AI)-assisted planning, augmented reality, and ablative procedures are expanding the technical repertoire [65, 66]. This review clearly demonstrates that robotic surgery should be viewed as a complement to, rather than a replacement for, open surgery. This is also in line with the international consensus: While the use of robotics is becoming possible in an increasing number of indications and perioperative morbidity can be reduced, there are still clear limitations in complex situations, such as multivisceral infiltrations, peritoneal carcinomatosis, or vascular infiltrations, in accordance with the principle that “not everything that is oncologically necessary can be performed using minimally invasive techniques.”

## Conclusions

In summary, the current developments outlined above underscore that abdominal surgery remains indispensable in gynecologic oncology. However, it is no longer defined primarily by one-dimensional radical resection, but rather by precise, risk- and biology-adapted interventions that are embedded in a complex multimodal therapeutic system.

The key aspects include the following points:

- Open surgery remains an important standard procedure in gynecologic oncology.
- Minimally invasive and robotic procedures expand the spectrum but do not replace open surgery.
- Robotics should be considered a complement, not replacement.
- Abdominal surgery is developing into an integrated, personalized, and technologically supported key component of modern oncological treatment strategies.

## Data Availability

No datasets were generated or analysed during the current study.

## Abbreviations

ABRAX: ABandoning RAdical hysterectomy in cerviX cancer
AI: Artificial Intelligence
AWMF: Arbeitsgemeinschaft der Wissenschaftlichen Medizinischen Fachgesellschaften
CC-0: Complete Cytoreduction (no macroscopic residual tumor)
CI: Confidence Interval
CHORUS: Primary chemotherapy versus primary surgery for newly diagnosed advanced ovarian cancer
ctDNA: Circulating Tumor DNA
DNA: Deoxyribonucleic Acid
EORTC: European Organisation for Research and Treatment of Cancer
FDA: U.S. Food and Drug Administration
FIGO: Fédération Internationale de Gynécologie et d’Obstétrique
GOG LAP 2: Gynecologic Oncology Group Laparoscopy Study 2
HR: Hazard Ratio
IDS: Interval Debulking Surgery
INTERLACE: Induction chemotherapy followed by standard chemoradiotherapy versus standard chemoradiotherapy alone in patients with locally advanced cervical cancer
LACC: Laparoscopic Approach to Cervical Cancer
LOS: Length of Stay
LVSI: Lymphovascular Space Invasion
MIS: Minimally Invasive Surgery
MRD: Minimal Residual Disease
NCCN: National Comprehensive Cancer Network
OS: Overall Survival
PARP: Poly (ADP-ribose) Polymerase
PD-1: Programmed Cell Death Protein 1
PD-L1: Programmed Death-Ligand 1
PDS: Primary Debulking Surgery
PFS: Progression-Free Survival
R0: Complete Resection (no residual tumor)
RCT: Randomized Controlled Trial
ROC: Recurrent Ovarian Cancer
SDS: Secondary Debulking Surgery
SHAPE: Simple Hysterectomy and Pelvic Node Assessment
SGO: Society of Gynecologic Oncology
SLN: Sentinel Lymph Node
TRUST: Trial of Radical Upfront Surgical Therapy
AGO: Arbeitsgemeinschaft Gynäkologische Onkologie (Working Group on Gynecologic Oncology)
AtTEnd: Atezolizumab Trial in Endometrial Cancer
CHIPOR: Chimio-Hyperthermie Intra-Péritonéale d’Ovaires en Récidive (Intraperitoneal Hyperthermic Chemotherapy for Recurrent Ovaries)
CRS: Cytoreductive Surgery
DESKTOP III: Descriptive Evaluation of preoperative Selection KriTeria for Operability in recurrent Ovarian cancer III
dMMR: Mismatch Repair-Deficient
DUO-E: Durvalumab and Olaparib in Endometrial Cancer
ECOG: Eastern Cooperative Oncology Group
ENGOT: European Network of Gynaecological Oncological Trial groups
HIPEC: Hyperthermic Intraperitoneal Chemotherapy
HORSE/MITO-18: Hyperthermic Intraperitoneal Chemotherapy in Platinum-Sensitive Recurrent Ovarian Cancer / Multicenter Italian Trials in Ovarian Cancer
LACE: Laparoscopic Approach to Carcinoma of the Endometrium
LANCE: Laparoscopic versus Open Surgery for Interval Debulking in Ovarian Cancer
LION: Lymphadenectomy in Ovarian Neoplasms
MIRRORS: Minimally Invasive Robotic Resection of Ovarian cancer after chemotherapy Study
MMR: Mismatch Repair
MSI-H: Microsatellite Instability-High
NRG-GY018: NRG Oncology Gynecologic Oncology Group Protocol GY018
NSMP: No Specific Molecular Profile
OVHIPEC-1: Ovarian Hyperthermic Intraperitoneal Chemotherapy-1
pMMR: Mismatch Repair-Proficient
POLE: Polymerase Epsilon (exonuclease domain mutation)
RUBY: GOG-3031/ENGOT-EN6 trial of dostarlimab in endometrial cancer
SCORPION: Surgical Complications Overalled Randomized Protocol in Interval versus Optimal Neo-adjuvant treatment
TCGA: The Cancer Genome Atlas
